# Serological evidence of Rift Valley fever virus infection among domestic ruminant herds in Uganda

**DOI:** 10.1186/s12917-021-02867-0

**Published:** 2021-04-13

**Authors:** Deo B. Ndumu, Barnabas Bakamutumaho, Edward Miller, Jesca Nakayima, Robert Downing, Stephen Balinandi, Fred Monje, Dan Tumusiime, Mary Nanfuka, Natascha Meunier, Eugene Arinaitwe, Chris Rutebarika, Eugene Kidega, Jackson Kyondo, Rose Ademun, Kariuki M. Njenga, Francisco Veas, Jean-Paul Gonzalez

**Affiliations:** 1 Department of Animal Health, Ministry of Agriculture, Animal Industry and Fisheries (MAAIF), P. O. Box 513, Entebbe, Uganda; 2 Uganda National Health Research Organization (UNHRO), Uganda Virus Research Institute (UVRI), P. O. Box 49, Entebbe, Uganda; 3 Metabiota Inc., San Francisco, USA; 4 National Livestock Resources Research Institute (NaLiRRI), Nakyesasa, Uganda; 5 Centers for Disease Control and Prevention (CDC) – UVRI, Entebbe, Uganda; 6 Royal Veterinary College, University of London, Royal College Street, London, NW1 0TU UK; 7 Africa Biomedical Laboratories, Nairobi, Kenya; 8 Molecular Comparative Immuno-Physiopathology Lab (LIPMC), Joint Research Unit-Ministry of Defense (UMR-MD), Faculty of Pharmacy, French Research Institute for Development (IRD), Montpellier University, 34093 Montpellier, France; 9 Georgetown University, School of Medicine, 3900 Reservoir Rd. NW, Washington, DC, 20007 USA; 10 Centaurus Biotech LLC., Commonwealth Trading Partners, CTP Inc. Alexandria, Virginia, USA

**Keywords:** Rift Valley fever virus, Sero-surveillance, ELISA, Epidemics

## Abstract

**Background:**

Prior to the first recorded outbreak of Rift Valley fever (RVF) in Uganda, in March 2016, earlier studies done until the 1970’s indicated the presence of the RVF virus (RVFV) in the country, without any recorded outbreaks in either man or animals. While severe outbreaks of RVF occurred in the neighboring countries, none were reported in Uganda despite forecasts that placed some parts of Uganda at similar risk.

The Ministry of Agriculture, Animal Industry and Fisheries (MAAIF) undertook studies to determine the RVF sero-prevalence in risk prone areas.

Three datasets from cattle sheep and goats were obtained; one from retrospective samples collected in 2010–2011 from the northern region; the second from the western region in 2013 while the third was from a cross-sectional survey done in 2016 in the south-western region. Laboratory analysis involved the use of the Enzyme Linked Immunosorbent Assays (ELISA). Data were subjected to descriptive statistical analyses, including non-parametric chi-square tests for comparisons between districts and species in the regions.

**Results:**

During the Yellow Fever outbreak investigation of 2010–2011 in the northern region, a total sero-prevalence of 6.7% was obtained for anti RVFV reacting antibodies (IgG and IgM) among the domestic ruminant population. The 2013 sero-survey in the western region showed a prevalence of 18.6% in cattle and 2.3% in small ruminants. The 2016 sero-survey in the districts of Kabale, Kanungu, Kasese, Kisoro and Rubirizi, in the south-western region, had the respective district RVF sero-prevalence of 16.0, 2.1, 0.8, 15.1and 2.7% among the domestic ruminants combined for this region; bovines exhibited the highest cumulative sero-prevalence of 15.2%, compared to 5.3 and 4.0% respectively for sheep and goats per species for the region.

**Conclusions:**

The absence of apparent outbreaks in Uganda, despite neighboring enzootic areas, having minimal restrictions to the exchange of livestock and their products across borders, suggest an unexpected RVF activity in the study areas that needs to be unraveled. Therefore, more in-depth studies are planned to mitigate the risk of an overt RVF outbreak in humans and animals as has occurred in neighboring countries.

## Background

Rift Valley fever (RVF) is a sub-acute to acute arthropod borne viral zoonotic disease, whose etiological agent is a ribonucleic acid (RNA) virus of the order *Bunyavirales*, family *Phenuiviridae* (the previous taxonomic division is *Bunyaviridae*) and of the genus *Phlebovirus* [1].

RVF was first described in 1910 and 1912, among exotic lambs in the Kenyan Rift Valley (between Lake Naivasha and Lake Elementaita). The virus was isolated and recognized as the etiological agent of the disease in 1931 [2, 3]. Later on, in 1944, K.C. Smithburn, working at the laboratories of the then Yellow Fever Research Institute in Entebbe, Uganda, obtained two virus isolates from mosquitoes of *Aedes tarsalis* and the *Eretmapodites* spp*.*collected in uninhabited forest in Western Uganda [4] from which isolates the Smithburn modified live virus vaccine (SMLVV) was derived [5, 6].

Before the disease emerged in Egypt in 1977, with severe epizootics in humans and animals [7], RVF was known to occur only in Eastern and Southern Africa. In 1987, RVF occurred for the first time in West Africa (in Mauritania) as a large and severe epizootic [8]. Further, in 1979, the disease emerged on the island of Madagascar [9] as well on the Arabian Peninsula in 2001 [10]. This dramatic extension was hypothesized to be associated with the trade routes and movements of viraemic animals [11].

In humans, RVF presents as an influenza-like illness that can evolve to severe hemorrhagic or neurological syndromes, [12]; more than 80% of infected people appear asymptomatic or present an influenza-like disease. In animals, RVF may occur in an epizootic form, over large areas following heavy rains with sustained flooding and is characterized by abortion storms, neonatal mortality and hepatitis, primarily in sheep, goats and cattle [13].

In Uganda, before the first recorded outbreak of RVF in March 2016, studies have indicated past serologic evidence of RVFV circulation [14–18] without any recorded overt outbreaks in humans or animals.

Neighboring countries in the Eastern African region, periodically experience large and severe RVF outbreaks such as the one of 1997–1998 that occurred in Kenya, Somalia, and Tanzania affecting over 100,000 people with over 450 deaths in Kenya alone [19, 20]. The RVF outbreaks spanning the period of December 2006 to June 2007, in the same Eastern African region, occurred in the neighboring countries of Kenya and Tanzania as well as Somalia and Sudan. Although, the outbreak was characterized by seven sequential outbreak foci; three in Kenya, two in Tanzania and two in Somalia, no RVF activity was reported, at the time, in Uganda despite its regional proximity to the affected countries and in spite of the early warning system developed by the USA National Aeronautics and Space Administration (NASA) that accurately predicted the latter outbreak as well as the outbreak areas. This same prediction indicated that Uganda was vulnerable and equally prone to RVF outbreaks, particularly so in the north eastern region [21, 22].

This paper examines three different sets of data with the aim of illuminating the serological status of domestic ruminant populations (cattle, goats and sheep) due to possible exposure to the RVF Phlevovirus, in the absence of overt outbreaks, in high-risk areas of northern, western and south-western regions of Uganda.

## Results

The first dataset of 75 sera specimens collected from cattle and small ruminants obtained during the investigation of a Yellow Fever outbreak of 2010/2011 in Agago and Kitgum districts (northern Uganda) showed an overall RVF antibody sero-prevalence of 6.7%; species sero-prevalences in this region were 4.7% in cattle and 9.4% in small ruminants for the two districts combined; both IgG and IgM antibodies to the RVF virus were detected at the time. However, only one sample had anti RVFV IgM antibodies (Table 1).

**Table 1:** Rift Valley fever sero-prevalence (on IgM, IgG ELISA tests) in cattle and small ruminants observed during a Yellow Fever outbreak investigation in Agago and Kitgum Districts in the northern region of Uganda (November 2010 to early 2011); ^a^One goat from Kitgum district tested positive for anti RVFV IgM antibodies

Species	District	positive / total tested (%)	95% CI
Cattle	Agago	1/37 (2.7)	0.0–14.2
	Kitgum	1/6 (16.7)	0.4–64.1
Sub Total		2/43 (4.7)	0.6–15.8
Small ruminants	Agago	1/3 (33.3)	0.8–90.6
	Kitgum	2/29 (6.9)^a^	0.8–22.8
Sub Total		3/32 (9.40)	2.0–25.0
Grand total	Agago	2/40 (5.0)	0.6–16.9
	Kitgum	3/35 (8.6)	1.8–23.1
Total		5/75 (6.7)	2.2–14.9

*Caption:* The chi-square test with Yates correction is not significant at *p* > 0.05 when comparing the two districts as well as comparison between cattle and small ruminants

The 2013 sero-survey specimens were collected from the domestic ruminant population in western Uganda from the districts of Hoima, Kibaale and Masindi; they were analyzed using an RVF inhibition ELISA test and showed a study sero-prevalence of 18.6% in the cattle population and 2.3% in the small ruminant population. District study sero-prevalences in cattle were respectively, 12.1, 10.0, and 25.0%, for the districts of Hoima, Kibaale and Masindi. In the small ruminant population, the district study sero-prevalence in Masindi was 3.1% while no antibodies were detected in Hoima district (Table 2).

**Table 2:** Rift Valley fever sero-prevalence (on IgG ELISA test) among cattle and small ruminants from the three Districts of Hoima, Kibaale and Masindi in Western Uganda (2013)

Species	District	positive / total tested (%)	95% CI
Cattle	Hoima	4/33 (12.1)	3.4–28.2
	Kibaale	2/20 (10.0)	1.2–31.7
	Masindi	15/60 (25.0)	14.7–37.9
Total cattle	3 districts	21/113 (18.6)	11.9–27.0
Small ruminants	Hoima	0/11 (0.0)	–
	Masindi	1/32 (3.1)	0.1–16.2
Total small ruminants	2 districts	1/43 (2.3)	0.1–12.3
Grand Total	2 districts + 2 species	23/156 (14.7)	9.6–21.3

*Caption.* The chi-square statistic test on cattle vs small ruminants is 4.0687, with a p-value of 0.043684 showing a significant (*p* < 0.05) difference between the two population

After the March 2016 RVF outbreak in Kabale, a planned multi-sectoral bio-surveillance pilot study (sero survey) was conducted in south-western Uganda including the outbreak district of Kabale and the four surrounding ones of Kanungu, Kasese, Kisoro and Rubirizi which showed the respective percentage of RVF reacting IgG antibodies of 16.0, 2.1, 0.8, 15.1 and 2.7% (Table 3).

**Table 3:** Rift Valley Fever sero-prevalence (on IgG ELISA test. Showing ‘positive / total tested (%)’) among cattle and small ruminants from five districts (Kabale, Kanungu, Kasese, Kisoro and Rubirizi) in south-western Uganda (2016)

District	Cattle		Goats		Sheep		Total	
	n/N (%)	95% CI	n/N (%)	95% CI	n/N (%)	95% CI	n/N (%)	95% CI
Kabale	66/280 (23.6)^a^	18.7–29.0	13/190 (6.8)	3.7–11.4	4/50 (8.0)	2.2–19.2	83/520 (16.0)	12.9–19.4
Kanungu	3/112 (2.7)	0.6–7.6	1/81 (1.2)	0.0–6.7	0/0 (0.0)	–	4/193 (2.1)	0.6–5.2
Kasese	1/69 (1.4)	0.0–7.8	0/60 (0/0)	–	0/1 (0.0)	–	1/130 (0.8)	0.0–4.2
Kisoro	17/67 (25.4)	15.5–37.5	4/52 (7.7)	2.1–18.5	0/20 (0.0)	–	21/139 (15.1)	9.6–22.2
Rubirizi	4/74 (5.4)	1.5–13.3	0/71 (0.0)	–	0/5 (0/0)	–	4/148 (2.7)	0.7–6.8
Total	91/600 (15.2)	12.4–18.3	18/454 (4.0)	2.4–6.2	4/76 (5.3)	1.5–12.9	113/1130 (10.0)	8.3–11.9

Legend: ^a^positive / Total tested (percent sero-prevalence)

*Caption.* The chi-square with Yates correction, cattle to goat: Chi-square = 34.9763. *p* < 0.00001. Highly significant at *p* < 0.01; Cattle to sheep: Chi-square = 5.4776. *p*-value = 0.019262. Significant at *p* < 0.05.; Cattle to goat + sheep: Chi-square = 37.943. *p* < 0.00001. Highly significant at p < 0.01; Goat to sheep: not significant *p* > 0.6; Comparison of districts close to Rwanda andthose further away (in this case Kabale, versus Rubirizi) in the south western region: Chi-square = 27.5106. *p* < .00001. Highly Significant at *p* < 0.01.)

In south-western Uganda, the districts of Kabale and Kisoro registered the highest district sero-prevalence above 15% in the domestic ruminant populations while Rubirizi, Kanungu and Kasese districts had a sero-prevalence lower than 3%. Of the three species investigated, bovines exhibited the highest cumulative sero-prevalence reaching 15.2%, followed by ovines and caprines with 5.3 and 4.0% respectively.

## Discussion

The three datasets provide recent serological evidence of possible RVFV circulation in selected areas of Uganda where anti RVFV antibodies were found in both cattle and small ruminant populations.

The pilot study of 2016 constitutes an initial multi-sectoral attempt to unveil the RVF sero-prevalence in a high-risk regions in Uganda, among domestic ruminant populations.

On a Chi-Square test comparison (with a Yates correction) among species, cattle from western and south-western regions had significantly higher anti-RVFV antibodies compared to goats and / or sheep. This can partly be explained by the longer life span of cattle compared to small ruminants with shorter life span. Additionally, the disproportionate sampling between the two species, particularly in the outbreak district of Kabale may have contributed to higher sero-prevalence in cattle. There was no significant difference between the goat and sheep populations sampled.

On the other hand, in the northern region, cattle sampled had lower anti-RVFV antibody sero-prevalence compared to small ruminants, but this was found not to be significant using the Chi-Square test.

The majority of the general sampling, from the northern region, gave negative results of IgM detection; only one test was found positive in a goat sample from the Kitgum district showing a recent infection and indicates a recent active transmission of RVF 6 to 8 weeks before our sampling, in the area or bordering areas.

The datasets from the three regions clearly reveal RVF virus reacting antibodies in susceptible hosts, it can be inferred that there has been previous, as well as ‘currently’ active, circulation of the virus within the domestic ruminant populations in the northern region of the country. The latter, however, cannot be inferred with certainty for datasets 2 and 3 obtained from the western and south western regions as anti RVFV IgM antibody data was not readily available.

It is clear that the domestic ruminant populations studied are not necessarily naïve to RVFV infection and it is, therefore, plausible to hypothesize that there is an unexplained transmission of the RVFV, probably mimicking disease occurrence during an ‘inter-epizootic’ period as is the case in the semi-arid regions which periodically experience large and severe outbreaks.

In the south-western region, the districts neighboring Rwanda had significantly higher anti-RVFV antibody sero-prevalence (*p* < 0.01) in the domestic ruminant population compared to the others; considering the case of Kabale vs Rubirizi, this can be partly explained by the prevailing eco-climatic condition as well as the socio-economic activities in the most southern districts (Kabale and Kisoro) which topographically, are characterized by steep hills and valleys; climatically Kabale district has a higher annual rainfall with a tendency of flooding; socioeconomically, there is trade in livestock and livestock products between Uganda and Rwanda. Whereas the more northerly districts (in the south-western region including Rubirizi) tend to be warmer, have lower annual rainfall and livestock trade is skewed in such a way that movement (of livestock and livestock products) tends to be more outwards from these areas to the eastern Democratic Republic of Congo (DRC).

The above trend in livestock trade notwithstanding, similar circumstances of serological evidence of RVF virus circulation in neighboring countries have been reported in the eastern Democratic Republic of Congo (DRC) in cattle using the ELISA assay [23]. The study areas included the provinces of North Kivu, South Kivu, and Ituri. The North Kivu province which borders much of western Uganda had the respective combined prevalence of anti-RVF IgG and IgM of 12.67 and 2%. In Rwanda, comparatively higher combined sero-prevalence of 16.8% in cattle were reported [24] although the Nyagatare district neighboring Uganda had a comparable seroprevalence of 7.9% in cattle.

Varying Rift Valley Fever sero-prevalences, in small ruminants, were reported in the DRC [25]; the study covered 15 territories within 7 provinces with the respective combined sero-prevalences of 0 to 23.81%, and 0 to 37.11% for goats and sheep. 12 out of 15 territories had seroprevalences of < 10% and some of these territories had comparable sero-prevalences reported in this study.

It is worth noting that in the above 3 studies (2 in DRC and 1 in Rwanda), there were no reported large and severe outbreaks, as was the case before the first outbreak in Uganda in March 2016.

It is also important to note that Uganda, Rwanda and Eastern DRC are largely part of the same African Tropical Pluvial-seasonal bioclimate with a number of limited areas of Tropical Xeric climate. The area is covered by deciduous forests, covering appreciable areas; bush-grass savannah in the northern region of Uganda and Mountain forest are shared by the 3 countries (mostly for the south-western part of Uganda). Altogether this shows an ecosystem unity, there are increasing changes in land cover and land use.

Consequently, this ecosystem unity can only partly explain the similarity of sero-prevalence as well as the reported circulation of RVFV; this is in view of the notion that transmissibility and spread of the disease are thought to be multi-factorial involving risk factors related not only to ecology but also climate, the vector and mechanical transmitters as well as socio-economic factors such as trade and cultural practices [21, 26, 27].

Depending on risk analysis studies, the cyclic nature of the disease and in consideration of the severity of outbreaks elsewhere there is need for preparedness plans to be setup.

Besides, it is also documented that despite the ‘quiescence’ of the RVFV without overt outbreaks, there is a possibility of large and severe outbreaks occurring; this is corroborated through a study in Malagasy, where severe outbreaks occurred in 2008 and 2009 in livestock and humans following two successive rainy seasons [9]. It would, therefore, be prudent for the Veterinary Services in Uganda to prepare contingency plans if ever the ‘right’ conditions should trigger an overt and large outbreak.

The studies in this paper also point out the need to characterize the RVFV potential variants which would possibly circulate at low noise without being detected in Uganda. Although the RVFV isolates belong to a particular serotype they are known to differ in virulence [28]; therefore, in this regard and given the non-overt manifestation of the RVFV in the study areas; a better understanding of the molecular epidemiology of the Ugandan isolates is highly warranted and should be determined along with the associated phenotype. Moreover, a report on the laboratory findings after the first reported RVF outbreak in Uganda [29], indicates that in 3 clinical specimens for which whole genome sequencing was done, the phylogenetic analysis inferred that the RVF Phlebovirus that was involved in the Kabale 2016 outbreak shows relatedness to the 2006–2007 RVF outbreak in Kenya.

## Conclusion

The presence of anti-RVFV IgG, and a singular case of IgM, antibodies in susceptible hosts, indicate a possible but often hidden circulation (i.e. limited IgM response) of the RVFV within the domestic ruminant populations with possible undetected (false negative diagnoses) or unreported outbreaks. Therefore, there is a need for improved capacity to detect disease outbreaks in livestock at very early stages by the National Veterinary Services as well as to improve bio-surveillance and reporting and to extend such bio-surveillance to epizootic and zoonotic diseases in the country. This should also be coupled with predictive modeling as indicated by the most recent dataset obtained from a bio-surveillance cross sectional study and prompted by the first two studies. More in-depth studies are already being designed aiming to mitigate the risk of RVFV transmission to both humans and animals, as well as to characterize the origin (phylogeography), genotype (possible reassortment) and phenotypes (pathogenicity) of the RVFV strains potentially present in Uganda.

## Methods

### Method and study design

#### Study sites

**Fig. 1:**
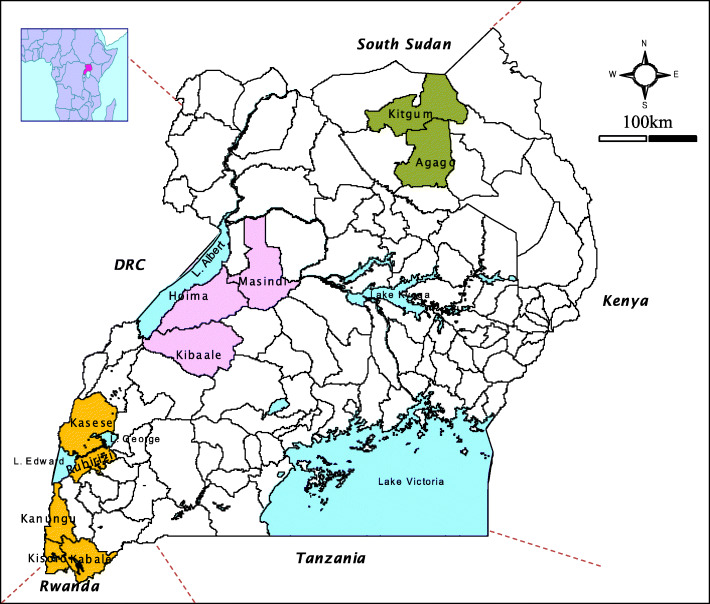
Uganda Map showing districts with suspected RVF endemic areas in domestic ruminants. Legend: Color coded Blue = lakes; Green = 2010 / 2011 RVFV reacting antibodies detected; Orange = 2016 RVFV reacting antibodies detected; Purple = 2013 RVFV reacting antibodies detected; white = non-tested districts. *Source; this map was obtained from the Uganda Ministry of Agriculture, Animal Industry and Fisheries (MAAIF) and drawn using the ArcView GIS version 3.2 software*

The districts in the 3 different regions in the country from which domestic ruminant samples were collected are shown in Fig. 1.

Retrospective and prospective sample analysis for RVFV reacting antibodies were carried out on the following sample sets, including: Set 1/ Samples were obtained during a yellow fever outbreak investigation based on syndromes during December 2010 to early 2011 in Agago and Kitgum districts; northern region. Sera were collected from cattle and small ruminants. Set 2/ Sampling from a cattle, sheep and goats sero-surveillance done in October 2013, in the districts of Hoima, Kibaale and Masindi; western region. Set 3/ A multi-sectoral bio-surveillance pilot study undertaken in the months of August to October 2016 in the RVF outbreak district of Kabale as well as in the surrounding districts of Kanungu, Kisoro and Rubirizi; south-western region.

### Description of study sites

The south-western districts of Kabale, Kisoro, Kanungu and Rubirizi have a montane climate with bimodal rainfall pattern; with peaks during the months of March to May and September to November. The mean annual rainfall ranges from 1000 mm to 1480 mm with temperature range of 10^0^ C to 27^0^ C. However, Kanungu and Rubirizi tend to be warmer and have lower rainfall compared to Kabale and Kisoro.

These districts are located in the Western Rift Valley system; the whole of Rubirizi and part of Kanungu districts are on the Western Rift Valley escarpment; the districts have a relief of between 1200 and 3000 m above sea level. The districts can be described as mainly green with interlocking hills and stretches of valleys. The vegetation is characterized by tropical forests including Bwindi Impenetrable and Echuya forests; there are coniferous plantations of Cyprus and pines trees in forest reserves, Eucalyptus tree plantations and woodland are a common feature. The region has water bodies and vast wetlands (swamps and marshland) associated with Lake Bunyonyi. Like the rest of the country, agriculture is the major form of livelihood. It relies on the naturally fertile volcanic and peat soils. This region is well known for production of horticultural crops. The hilltops and valleys are also used for livestock production; other agricultural ventures include the growing of cash crops (coffee, tea, pyrethrum). The region is also well known for diary production, agro-forestry and apiary.

The district of Kasese is also within the Western Rift Valley, it is much drier with a wooded savannah vegetation on gentle rolling hills. The foothills of Mt. Rwenzori are appreciably forested.

The two northern Uganda districts have a bush grassland savannah, several water bodies and swamps with a bimodal rainfall pattern of between 800 mm and 1200 mm annually. The Western region districts of Kibaale, Hoima and Masindi are characterized by tropical rain forests.

The mean annual rainfall ranges from 1000 mm to 1300 mm with a temperature range of 17 °C to 28 °C.

In the 3 regions there are increasing changes in land use and therefore, land cover.

### Selection of animals for the three study datasets

For the retrospective study in northern Uganda (dataset 1), syndromic surveillance was applied. Animals were selected by way of a non-probabilistic risk-based approach. Human medical samples for pathogen detection led to confirmed human cases of Yellow Fever. The samples (serum) from the domestic ruminant population (cattle, sheep and goats) had antibodies to RVFV.

Dataset 2 was part of the routine Ministry of Agriculture Animal Industry and Fisheries (MAAIF) sero-surveillance for RVF, animals in this dataset were part of a preselected sample based on multistage sampling; while dataset 3 was derived from a planned cross-sectional study where animals were selected also based on a multistage sampling design;

The approach used to select the total sample size involved selection of a region in a high risk area, namely, south-western Uganda; farms were selected from villages in the outbreak district and the surrounding ones. The minimum number of animals, irrespective of species, were randomly selected basing on the formula indicated here below [30].

$$ {\mathrm{n}}_{\mathrm{min}}= DE\times \frac{Z^2\times p\times \left(1-p\right)}{d^2} $$

n_min_ = minimum sample size of domestic ruminants (cattle, sheep and goats) (1153); DE = Design Effect (3); d = acceptable error margin (0.05); Z = Z score (for a 95% confidence interval); *p* = Assumed prevalence of the disease in the population (0.5).

#### Sample collection

Sera samples were collected from domestic ruminants (cattle, goats and sheep), preserved and transported along a cold chain following the standard operating procedures (SOPs) approved at the National Veterinary Laboratory of the MAAIF (National Veterinary Referral Laboratory).

A total of 1361 samples were tested, including 75 sera samples from the 2010 / 2011 Yellow Fever outbreak investigation, 156 sera samples from a routine RVF Sero-survey (2013) and 1130 sera samples from the 2016 RVF Pilot Study supported by the Defense Threat Reduction Agency (DTRA), Cooperative Biological Engagement Program CBEP [31].

All sera specimens from all districts sampled were tested using the ELISA and showed RVFV reacting antibodies among the domestic ruminant populations. Data were analyzed and the threshold of statistical significance was stated at *p*-value < 0.05.

### Laboratory sample analyses

#### Sample set 1

Laboratory tests were performed in the Centers for Disease Control and Prevention (CDC) laboratory at Kenya Medical Research Institute (KEMR) Laboratory, Kenya, using an in-house direct immunoglobulin G (IgG) detection by Enzyme Linked Immunosorbent Assay (ELISA) as well as the immunoglobulin M (IgM) capture ELISA. Both IgG and IgM antibodies were detected. Only one sample exhibited anti RVFV IgM antibodies.

#### Sample set 2

Samples were analyzed using RVF inhibition ELISA at the National Veterinary Laboratory of the National Animal Disease Diagnostic and Epidemiology Centre; no tests were done for anti RVFV IgM antibodies.

#### Sample set 3

The sera specimens were analyzed using an in-house direct IgG antibody ELISA at the CDC Uganda Virus Research Institute (UVRI) laboratories. All sera specimens were tested in the lab using Enzyme Linked Immunosorbent Assay (ELISA) for anti-RVFV IgG antibodies only, no tests were done for anti RVFV IgM antibodies.

The laboratory test results of sample sets 1 and 3 were obtained using the CDC serological test protocols that classify a specimen as positive meeting 2 pre-established and conservative criteria; namely OD value at 1/400 titer must be > 0.2; and its OD Sum must be > 0.95. These criteria have been established over time and have high reproducibility of the results. Thereby, taking care of the lower limit of detection with respect to titter values that are set at 1/400.

The laboratory test results of datasets 2 were obtained using an inhibition Enzyme-linked immunsorbent oassay (ELISA) for the detection of antibodies to Rift Valley fever virus in humans, domestic and wild ruminants developed at the National Institute for Communicable diseases [32].

The lower control limits for net OD for the positive control and negative control were respectively minus 0.05 and 0.65; whereas the same values for the upper control limits were 0.07 and 1.34; the test in terms of Percent Inhibition (PI) for the positive control and negative control were, respectively, 94.26 and 4.26% for the lower control limits; whereas for the upper control limits corresponding values were 102.8 and 4.33%; while the diagnostic sensitivity and specificity were respectively 99.47% PI and 99.66% PI.

### Data analysis

The data presented from the 3 datasets were principally observational, consequently unadjusted descriptive statistical analyses were employed including the computation of proportions to derive study sero-prevalences;

The data were captured, collated, analyzed using MS Excel® software spreadsheet in Microsoft office for dataset 3 and then annotated. The descriptive statistics and confidence intervals were calculated in R Statistical Software using the package *binom* [33]..

For each dataset a non-parametric chi-square test with Yates correction was used to do paired comparison between districts and species (cattle, sheep and goats) within each region for the 3 regions. The district comparisons were done for only datasets 1 and 3.

## Data Availability

All data and material of the present study are available upon request to the corresponding authors including all field and laboratory original data anonymized in an excel format.

## Abbreviations

ASM: American Society of Microbiology
CBEP: Collaborative Biological Engagement Program of DTRA
CDC: Centers for Disease Control and Prevention
CTP: Commonwealth Trading Partners
CVL: Central Veterinary Laboratory (of Kenya)
DTRA: Defense Threat Reduction Agency
ELISA: Enzyme Linked Immunosorbent Assay
GIS: Geographic Information System
IgG: Immunoglobulin type G
IgM: Immunoglobulin type M
IRD: French Research Institute for Development
KEMRI: Kenya Medical Research Institute
LIPMC: Molecular Comparative Immuno-Physiopathology Lab
MAAIF: Ministry of Agriculture, Animal Industry and Fisheries
NASA: National Aeronautics and Space Administration of the USA
OD: Optical Density
OIE: Office Internationale des Epizootes (World Organization for Animal Health)
PI: Percent Inhibition
REC: Research Ethics Committee
RVF: Rift Valley fever
RVFV: Rift valley fever virus
SMLVV: Smithburn modified live virus vaccine
SOP: Standard Operating Procedure
UMR-MD: Joint Research Unit-Ministry of Defense
UNHRO: Uganda National Health Research Organization
USA: United States of America
UVRI: Uganda Virus Research Institute
WHO: World Health Organization of the United Nations
